# Factors Affecting Response to Infertility Treatment: Case of Iran

**DOI:** 10.5539/gjhs.v8n1p118

**Published:** 2015-05-15

**Authors:** Fatemeh Peyromusavi, Mohsen Barouni, Tayebeh Naderi, Arash Shahravan

**Affiliations:** 1 Oral and Dental Diseases Research Center kerman University of Medical Sciences, Kerman, Iran; 2 Health Services Management Research Center, Institute for Futures Studies in Health, Kerman University of Medical Sciences, Kerman, Iran; 3 Kerman University of Medical Sciences, Kerman, Iran

**Keywords:** Response to treatment, infertility, menstruation, body mass index

## Abstract

**Introduction:**

Infertility affects both women and men in all the countries. Infertility often has profound long-term or short-term impacts on the people involved and puts them at risk of familial and social pressures. According to WHO estimates, between 8% and 12% of all the couples worldwide experience some form of infertility during their reproductive life, i.e. 50–80 million people are affected. The aim of this study was to evaluate the response to infertility treatment by taking into account factors such as age, hirsutism, menstruation and galactose among women in Kerman.

**Methodology:**

Of a total of 300 patient files evaluated 220 cases were flawless, of which the study factors were recorded. These data were estimated by Logit model. The dependent variable was the response to treatment (0 and 1) and the independent variables included age of men and women, hirsutism, menstruation, galactose, duration of the period no preventive measures were used and body mass index. After entering the data, model output was analyzed by using the STATA software.

**Results:**

The results showed that of all the model variables, female age (prob=0.0065), menstruation (prob=0.04), hirsutism (prob=0.02), marriage age (in months) (prob=0.02) and BMI were significant and other variables were not significant. McFadden analysis for goodness of fit was 0.92.

**Conclusion:**

The study results showed that women should pay more attention to variables such as BMI, menstruation quality (regular and irregular) and aging because clinical disregard of any of the above can have a significant impact on the individual’s fertility.

## 1. Introduction

Infertility is defined as the inability of a couple to conceive a child after one year of regular sexual life without contraceptive techniques (Tekin et al., 2004). Infertility is one of the major health-therapeutic problems in different societies. The high prevalence (8 to 12%) of this problem makes it very important. A significant proportion of infertility is dependent on environmental conditions and acquired risk factors which are preventable. Different environmental conditions show the necessity of studying the different causes of infertility in each region. In today’s modern world there are many reasons for infertility in men and women, one of the most important of which from the viewpoints of experts is the age of the couple. Couples should be aware that the fertility rate of a woman under 25 years of age is more than 80% but for a woman over 35 this rate is under 50% (Tekin et al., 2004).

It is likely that the number of ova is limited after reproductive age; that factor alone can reduce fertility and increase the risk of birth defects in the fetus and newborn. In addition, men over 35 compared with men under 35 have less chances of fertility, and the risk of chromosomal abnormalities is higher in their children. One of the factors with a significant impact on male infertility is environmental, industrial and air pollution, which minimizes the sperm counts, and decreasing fertility (Tekin et al., 2004).

According to studies, obesity and ovarian dysfunction in women are risk factors for infertility. Various studies conducted around the world have shown that women who are overweight or obese require more time to get pregnant, compared with normal or standard weight women, and pregnancy in obese women is more likely to be unsuccessful. According to experts, obesity in men and women increases the level of hormones in their bodies, affecting the reproductive process. In fact, abnormal secretion of hormones can lead to weight gain and negative effects on the male and female germ cell production (Tekin et al., 2004).

One of the most common causes of infertility is ovulation failure in women. It usually occurs due to malfunction of the hypothalamus, resulting in the pituitary gland dysfunction in women. Stress, trauma, excessive weight gain or loss, anorexia or eating disorder and vigorous exercise are factors that affect the activity of the ovaries. Therefore a review of the underlying diseases and also lifestyle modification and consultation with a gynecologist can be applied for the treatment and prevention of this disorder (Speroff & Fritz, 1999).

Studies have shown that a high percentage of women with hirsutism have ovulation dysfunction. Excessive hair growth in certain areas of the face and body in women is called hirsutism or excessive hairiness (Shah & Patel, 2009). Causes of infertility in women, in order of frequency, are: tubal disorders, lack of ovulation (disorders of the ovaries), cervical disorders, endometriosis and uterine causes. Half of all cases of infertility are due to ovulation dysfunction of the ovaries. Regular menstruation in women is a sign of proper ovulation and irregular menstrual cycles show there is no proper ovulation, which necessitates an investigation. The only certain sign of ovulation is pregnancy. The main treatment for infertile women with infertility due to ovulation disorders is to take fertility drugs. These drugs stimulate or regulate ovulation. In general, they act like natural hormones such as LH and FSH and activate the ovulation process (Raga, Bonilla-Musoles, Casan, & Bonilla, 1999).

The first and best line of treatment for ovulation induction is to use Clomiphene, and combination therapy often results in pregnancy in Clomiphene-resistant patients (Wolf, 2000).

The etiology of infertility and fertility patterns of different causes in different regions are different due to major differences in environmental conditions associated with reproductive behaviors, such as age at marriage, number of sexual partners, environmental pollution, alcohol consumption and smoking, and the prevalence of infectious diseases in different communities. These differences, particularly between developed and developing countries, are considerable. The major and most logical approach for reducing the problem of infertility is to try to reduce the incidence of infertility and improve reproductive health in order to prevent the incidence of infertility (Karim Pour et al., 2005). Knowledge about the different causes of infertility in every area is important from a healthcare point of view and can be effective in the management of decisions. The aim of this study was to evaluate the response to treatment of infertility by taking into account factors such as age, hirsutism, menstruation quality and galactose in women in Kerman in 2014.

## 2. Materials and Methods

The records of all the patients who had undergone ovulation induction procedure at the Clinic of Obstetrics and Gynecology, Kerman, were investigated. In all the patients, ultrasound examination of the follicle size and endometrial thickness had been performed on the day 14 of the menstrual cycle and in patients with 2 or more than 2 follicles larger than or equal to 18 mm (in case of treatment with Clomiphene, Bromocriptine, Dexamethasone, or a combination of these drugs) and those with 2 or more than 2 follicles larger than or equal to 16 mm (in case of treatment with HMG) and endometrial thickness of 6 mm or more, HCG had been injected intramuscularly at a dose of 10,000 units; the patient’s response to treatment was considered positive at this stage. A total of 220 out of 300 evaluated files compiled during the first 6 months of 2014 were flawless and the factors required for the purpose of this study were recorded. The data were estimated by Logit model. Logit model is used in cases in which the dependent variables appear in the form of a dichotomous choice. In logistic regression analysis, such as multivariate regression, coefficients of independent variables are estimated, but their functions are different. In multiple regressions analysis the sum of the least squares is used. This way, the sum of square differences between the actual and predicted values of the dependent variable will be minimal. The logistic model follows the logistic curve, so the curve is processed based on real data. Two values of ‘0’ and ‘1’ can be assigned to real data on the dependent variable based on the desired effect occurring or not occurring so that they are placed at the top or bottom of the related charts. The advantage of logistic regression is that to determine the values of ‘0’ and ‘1’ a notification of the occurrence of the phenomenon in question is sufficient. Using this model, the dependent variable was the response to treatment (0 and 1) and the independent variables included age of men and women, hirsutism, menstruation, galactose, the period of prevention and body mass index. After entering the data, the model output was analyzed using the software STATA.

The functional basic form of the model is as follows.

Equation (1): The functional form of the Logit model:


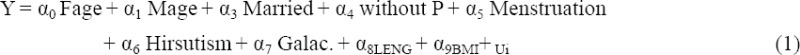


In this model Y is the dependent variable which indicates whether the treatment was successful or not as indicated by the value of ‘0’ or ‘1’. Fage is the age of the women under study; Mage is the age of the men under study; Married is the number of months elapsed after marriage; without P is the number of months without prevention; Menstruation indicates the regularity or irregularity of menstrual cycles of the women under study, defined as Dummy variable in the model; Hirsutism represents presence or absence of excessive hairiness; Galac shows normal or abnormal secretion; LENG shows duration of infertility treatment; and finally, BMI indicates body mass index. In every model, Ui represents the remaining sentences of the model.

It has been explained in different methodology sources for the Logit model that in order to validate the results of the model there should exist at least 10 samples per each independent variable (10). In this model we had to include 90 samples which is the minimum of the samples but a sample of 220 was selected, which is also valid. Initially the records (as census) were considered by researchers, but because there was lack of information in some cases only 220 flawless cases were included. It should be noted that the coefficients of the Dummy variables in the Logit models (0 and 1) are considered as the false coefficients. The final effect of these variables must be considered, and these were calculated using STATA software. In case of any estimated model, goodness of fit of model was estimated by Hosmer-Lemeshow test. This test compares the expected fitted values with the actual values for each category or group by data clustering. The important thing is that significant variables influencing the model were determined after model specification. Anisotropy of the variance in the studied models was tested by using Davidson and MacKinnon dummy regression method and the null hypothesis of no anisotropy in the variance of each of the models was accepted.

## 3. Results

Of 220 cases of infertility, the females were 17 to 44 years of age, with a sample size of 17 patients (2.7%) over 35, with only 5 patients exhibiting a positive response to treatment. It should be noted that in this study population, 76 cases were females under 25 years of age, of which 52 patients exhibited a positive response to treatment. In general, the basic descriptive statistics showed that 64 (29%) exhibited a positive response to treatment. Approximately 156 (71%) reported a positive response to treatment. The results showed that of 156 patients responding to treatment only 5 women were over 35 years of age.

Variables associated with the menstrual cycle showed that of 220 patients, 80 had regular menstrual cycles (3.36%), with the rest (7.63%) having irregular menstrual cycles.

Variables associated with hirsutism showed that of the 220 subjects, 61 (27.7%) were positive for hirsutism, meaning these people suffered from hirsutism in certain parts of the body and the rest (72.27%) were negative for hirsutism. Also, 40 cases (18.18% of the total sample and 65.57% of positive hirsutism samples) of the 61 positive hirsutism samples, had irregular menstruation; in addition, 21 cases (9.5%) of these patients exhibited positive responses to treatment.

Variables associated with galactose showed that of 220 subjects, 36 cases (16.36%) were positive for galactose; these subjects had secretions from the breasts, and in the rest (83.64%) galactose was negative. However, 21 cases (9.5% of all the samples and 58.33% of the 36 samples positive for galactose) of 36 galactose-positive samples had irregular menstrual cycles, of which only 11 samples (5%) responded to treatment positively. Statistics showed that 12 cases (5.45%) of all the samples had hirsutism and secretions from the breasts (galactose) at the same time, of which 7 (18.3%) patients responded to treatment positively.

Statistics associated with age of men showed that of 220 subjects, 27 (12.27%) were males over 38, 22 (10%) of whose wives responded to treatment positively; 25 (11.36%) were under 25 years of age, 14 (6.36%) of whose wives responded to treatment positively. The rest of the subjects (168, 76.36%) were in the 25to38 age group.

In relation to the duration of marriage, data showed that in 33 cases (15%) the marriage was less than 12 months old, of which 20 (9.09%) positively responded to treatment. In addition, in 61 subjects (27.73%) marriage was more than 60 months old (5 years), of which 43 (19.55%) exhibited a positive response to treatment. Thus, in 126 cases (57.27%) the marriages were 1 to 5 years old, of which 93 (42.27%) patients exhibited a positive response to therapy.

In relation to the number of months without contraceptive techniques, the results showed that in 51 cases (23.18) there were less than 12 months of attempts to conceive a child, of which 37 (16.82%) responded positively to treatment. In addition, in 17 cases (7.73%) there were 60 months of efforts for pregnancy, of which only 9 (4.1%) responded positively to treatment. There were 152 cases (69.1%) with 12 to 60 months without contraceptive techniques, of which 110 cases (50%) exhibited a positive response to therapy.

## 4. Discussion

In studies conducted by Speroff, male factor was responsible for 25 to 40% of infertilities in couples but the ovarian factor was responsible for infertility in women in 30 to 40% of the cases (Speroff & Fritz, 1999). Frank also reported that the most common reason for infertility in women in their study was polycystic ovary syndrome (PCOS) which affected a significant percentage of women with infertility (Frank, 1995). In the meantime, there are different methods for detecting ovulation; ultrasound is very accurate, but the method is expensive (Berek, 2007). In this study, ultrasound was used to confirm ovulation.

Nouhjah et al showed in a study on college students that hirsutism had a significant relationship with weight gain, menstruation disorders and a family history of hirsutism, with menstruation quality being reported as a variable affecting hirsutism (Nouhjah et al., 2011). In the present study, too, it was shown after examining the samples that 40 of the 61 cases positive for hirsutism also had irregular menstruation cycles; in addition, there was a relationship between menstruation cycle quality and response to the treatment of infertility (P=0.04) (Table 1). In addition, a relationship has been shown in other studies between hirsutism and obesity due to the conversion of hormones in adipose tissue (Azizi, 2007). Ghaderi et al. (2004) in a study in Birjand University of Medical Sciences showed the odds of hirsutism in obese and overweight subjects were 11.9 times more than subjects with normal BMI (Ghaderi, 2004). In another study conducted by Akhyani et al, the chance of hirsutism in women with abnormal menstrual patterns was 48.1 times more than that in women with normal patterns (Akhyani et al., 2006). Another study showed a higher prevalence of hirsutism in women with menstrual disorders (Tirgar Tabari, Haji Ahmadi, Gholi Nejad, & Talebzadeh Noori, 2002).

**Table 1:** Results of logit model on variables examined in the study

Dependent variable	[95% CI]		P-Value	Std. Err	Coefficients
Galactose	11.20442	-1.454242	0.131	3.229309	4.875087
Female Age	-0.1875079	-1.105623	0.006	0.2342172	-0.6465652
Hirsutism	10.80578	0.4872719	0.032	2.632321	5.646525
Menstruation	7.814959	-0.2642217	0.067	2.061053	3.775368
No of course of medic.	1.942261	-0.3949668	0.194	0.5962425	0.7736472
Duration of course of medic.	0.9979382	-4.218166	0.226	1.330663	-1.610114
Male Age	0.4246935	-0.0346065	0.096	0.1171705	0.1950435
Contraception	0.0054278	-0.1387388	0.070	0.0367779	-0.0666555
No. of months of marriage	0.1001661	0.0106568	0.015	0.0228344	0.554114
BMI	23.28047	2.865337	0.012	5.208038	13.0729
cons	16.90192	-1.54186	0.103	4.705132	7.680029

Logistic Regression Log likelihood=-11.390177; consNumber of obs = 220; LR chi^2^(10) = 242.52; Prob > chi^2^ = 0.0000; Pseudo R^2^ = 0.9141.

Other studies have shown that menstrual cycle disorders, infertility and increased prevalence of insulin resistance syndrome are the complications associated with hirsutism (Bochra et al., 2005; Kim et al., 2011). In the present study, too, the variables associated with hirsutism in the response to treatment in patients with infertility were significant (P=0.02). Variables associated with female age in studies conducted by Esmaeilzade (Esmaeil Zadeh, Jorsaraei, Farsi, Haji Ahmadi, & Rezaei, 2003) and Ale Yasin (Ale Yasin, Agha Hosseini, Khademi, & Saeidi Saeidabad, 2000) showed that fertility decreased with aging. In this study, the relationship between age of women and response to infertility treatment was significant (P=0.0065).

## 5. Conclusion

In this study, The Logit model was used in 220 cases of infertility, showing that among model variables, the variable of female age (prob=0.0065), menstrual cycle quality (prob=0.04), hirsutism (prob=0.02), the number of month into marriage (prob=0.02) and BMI were significant and other variables were not significant. McFadden analysis yielded a goodness of fit value of 0.92. Therefore, from these results we can suggest that women should pay more attention to variables such as BMI, menstrual cycles (regular and irregular) and aging because disregarding any of the above can have a significant impact on fertility.
